# Aberrant activation of *CYR61* enhancers in colorectal cancer development

**DOI:** 10.1186/s13046-019-1217-9

**Published:** 2019-05-22

**Authors:** Lingzhu Xie, Xuhong Song, Hao Lin, Zikai Chen, Qidong Li, Tangfei Guo, Tian Xu, Ting Su, Man Xu, Xiaolan Chang, Long-Kun Wang, Bin Liang, Dongyang Huang

**Affiliations:** 1 Department of Cell Biology and Genetics, Key Laboratory of Molecular Biology in High Cancer Incidence Coastal Chaoshan Area of Guangdong Higher Education Institutes, Shantou University Medical College, Shantou, 515041 China; 2 Department of Gastroenterology, Shantou Central Hospital, Shantou, 515041 China; 3 Department of Clinical Laboratory, Jiujiang First People’s Hospital, Jiujiang, 332000 China; 4 Department of Cell Biology and Genetics, Shantou University Medical College, Complex Building, Room 602, No. 22 Xinling Road, Shantou, Guangdong China

**Keywords:** CYR61, Enhancer, FOXA1, CBP, H3K27ac, Colorectal cancer

## Abstract

**Background:**

High expression of secreted matricellular protein cysteine-rich 61 (*CYR61*) correlates with poor prognosis in colorectal cancer (CRC). Aberrant enhancer activation has been shown to correlate with expression of key genes involved in cancer progression. However, such mechanisms in *CYR61* transcription regulation remain unexplored.

**Methods:**

Expression of *CYR61* was determined by immunohistochemistry (IHC), quantitative real-time PCR (qRT-PCR) and western blotting (WB) in CRC patients paraffin specimens and colon cell lines. ChIP-seq data of enhancer-characteristic histone modifications, in CRC tissues from the Gene Expression Omnibus (GEO) database, were reanalyzed to search for putative enhancers of *CYR61*. Dual-luciferase reporter assay was used to detected enhancer activity. Physical interactions between putative enhancers and CYR61 promoter were detected by chromosome conformation capture (3C) assay. Histone modification and transcription factors (TFs) enrichment were detected by ChIP-qPCR. Additionally, biological function of enhancers was investigated by transwell migration assays.

**Results:**

CRC tissues and cell lines expressed higher level of *CYR61* than normal colon mucosa. Three putative enhancers located downstream of *CYR61* were found in CRC tissues by ChIP-seq data reanalysis. Consistent with the ChIP-seq analysis results in the GEO database, the normal colon mucosal epithelial cell line NCM460 possessed no active *CYR61* enhancers, whereas colon cancer cells exhibited different patterns of active *CYR61* enhancers. HCT116 cells had an active Enhancer3, whereas RKO cells had both Enhancer1 and Enhancer3 active. Pioneer factor FOXA1 promoted *CYR61* expression by recruiting CBP histone acetyltransferase binding and increasing promoter-enhancer looping frequencies and enhancer activity. CBP knockdown attenuated H3K27ac enrichment, promoter-enhancer looping frequencies, and enhancer activity. Small molecule compound 12-O-tetradecanoyl phorbol-13-acetate (TPA) treatment, which stimulated *CYR61* expression, and verteporfin (VP) treatment, which inhibited *CYR61* expression, confirmed that the enhancers regulated *CYR61* expression. Knockdown and ectopic expression of CYR61 rescued cell migration changes induced by over-expressing and knockdown of FOXA1, respectively.

**Conclusions:**

*CYR61* enhancer activation, mediated by FOXA1 and CBP, occurs during CRC progression to up-regulate *CYR61* expression and promote cell migration in CRC, suggesting inhibition of recruitment of FOXA1 and/or CBP to *CYR61* enhancers may have therapeutic implications.

**Electronic supplementary material:**

The online version of this article (10.1186/s13046-019-1217-9) contains supplementary material, which is available to authorized users.

## Background

*CYR61* (cysteine-rich 61/CCN1) belongs to the CYR61/CTGF/NOV (CCN) protein family [1]. As a secreted matricellular protein, CYR61 can bind directly to various integrin receptors and heparan sulfate proteoglycans to regulate many cellular functions in a cell type- and context-dependent manner [1–3]. High expression of CYR61 is observed in colon cancer tissues and is closely related to shorter survival in colon cancer patients, and has been reported to promote cancer metastasis and cell migration [4–9]. Transcription factors (TFs), such as SOX4 [4] and FOXK1 [7], can up-regulate *CYR61* expression by binding to the *CYR61* promoter in colon cancer cells. However, the research of *CYR61* transcriptional regulation in CRC is limited.

Enhancers are important cis-regulatory elements and function as integrated TF docking platforms [10]. Dysregulation of enhancers induces aberrant gene expression that drives the uncontrolled proliferation of human cancers, including colon cancer [11, 12]. Generally, active enhancers are loaded with lineage-specific TFs (sequence-dependent) and coactivator proteins (which lack sequence-specific DNA-binding) [13]. Mediator complex, composed of coactivator proteins, can simultaneously bind to different TFs to mediate enhancer-promoter looping [14, 15] and facilitate delivery of important accessory factors to the promoter to potentiate transcription [16]. Enhancer RNAs (eRNAs), which are transcribed from the enhancers, are considered to be reliable markers for active enhancer activity [17, 18]. Epigenetic modifications, such as histone modifications, can influence enhancer activity, with some histone modifications being considered hallmarks of enhancers and enhancer activity [16]. In particular, DNA elements decorated with monomethylated H3 lysine 4 (H3K4me1) alone are considered to be primed enhancers and, when combinatorially deposited with acetylated H3 lysine 27 (H3K27ac), are considered to be active enhancers [16, 19].

CBP is closely related to its paralogue p300. As a co-activator, CBP can bind to TFs and bridge them to large protein complexes, such as Mediator complex [20]. Moreover, CBP can act as a lysine acetyltransferase to acetylate TFs and histones to increase the accessibility of chromatin [20, 21]. Forkhead box A1 (FOXA1) has been reported to be a pioneer factor in that it can bind to and open compacted chromatin to facilitate the binding of other TFs, and its activity is dysregulated in many physiological and pathological conditions [22, 23]. Other forkhead transcription factors, such as FOXK1 [7] and FOXO3a [24], have been reported to regulate *CYR61* expression by binding to the *CYR61* promoter in a sequence-dependent manner. Thus, FOXA1 might also regulate *CYR61* expression in a sequence-dependent manner.

Although enhancers play important roles in gene transcriptional regulation, the role of enhancers in regulating *CYR61* transcription in human colon cancer remains unexplored. By analyzing histone modification hallmarks of enhancers in colon cancer, we identified three putative enhancers located downstream of *CYR61*. In this study, we demonstrate that two of these three enhancers were aberrantly activated in CRC and, combined with FOXA1 and CBP, play key roles in the activation of *CYR61* expression in colorectal cancer.

## Methods

### Patients and tissues

A total of 42 cases of colonic adenocarcinoma (along with corresponding matched normal colonic mucosa), obtained from colon cancer patients who underwent surgical treatment from 2015 to 2016 at Shantou Central Hospital (Guangdong, China), were available for examination in this study. Tissues were embedded in paraffin. All collected tissues had pathologic diagnoses by two independent pathologists at the Shantou Central Hospital. Patients who received radiotherapy or chemotherapy prior to surgery were excluded.

### Immunohistochemistry (IHC)

Specimen slides were incubated with anti-CYR61 primary rabbit polyclonal antibody (1:200; ThermoFisher, PA116579) overnight at 4 °C. Anti-mouse/rabbit HRP-labeled secondary antibody (Maixin Biological Technology Co. Ltd., KIT-5010) was applied for 20 min at RT. Slides were then incubated with diaminobenzidine (DAB) chromogen substrate and counterstained with hematoxylin. Image-Pro Plus v.6.0 (IPP 6.0) software (Media Cybernetics, Inc., USA) was used to assess the area and the integrated optical density (IOD) value of the stained region. Mean density = IOD/area. The average mean density for five random fields at 100× magnification was used for CYR61 statistical analysis.

### Microarray analysis

The mRNA expression levels of *CYR61* in normal colonic samples and colon cancer samples were analyzed by ONCOMINE microarray datasets (https://www.oncomine.org), with a cut-off *p*-value of 0.001, and fold change of 2.0 [25, 26].

### Cell culture

The normal human colon mucosal epithelial cell line NCM460 (INCELL Corporation, USA) was cultured in M3D medium (INCELL Corporation, USA). The human colon carcinoma cell line LoVo (Cell Bank of the Chinese Academy of Sciences, China) was cultured in basic DMEM/F-12, HEPES (Gibco, USA); C2BBe1 [Caco-2 cell clone] (ATCC, USA) was cultured in DMEM (Gibco, USA) with 0.01 mg/ml human transferrin; the HCT116 cell line (Cell Bank of the Chinese Academy of Sciences, China) was cultured in McCoy’s 5A (modified) medium (McCoy’s 5A) (Gibco, USA); SW480 (ATCC, USA) was cultured in Leibovitz’s L-15 Medium (Gibco, USA); RKO (ATCC, USA) was cultured in minimum essential medium (MEM) (Gibco, USA). All cell culture media was supplemented with 10% fetal bovine serum. All cell lines were cultured in a humidified atmosphere at 37 °C. SW480 was cultured with atmospheric air, and other cell lines were incubated with 5% CO_2_.

### Compounds

Compounds used in this research were as follows: 12-O-tetradecanoyl phorbol-13-acetate (TPA) (Sigma, P8139) and verteporfin (VP) (Sigma, B1583–5).

### RNA extraction, RT-PCR and quantitative PCR

Total RNA was isolated using Trizol (Takara, No.9109) according to the manufacturer’s instructions. Reverse transcription was performed using a PrimeScript™ RT reagent Kit with gDNA Eraser (Takara, RR047A). Quantitative real-time PCR was performed using AceQ qPCR SYBR Green Master Mix (Low ROX Premixed) (Vazyme, Q131–02) on a QuantStudio 12 K Flex Real-Time PCR System (ThermoFisher, USA) according to the manufacturer’s protocols. Primers we designed are shown in Additional file 1: Table S1.

### Western blot assay (WB)

Antibodies used for western blot were as follows: anti-CYR61 (CST, #14479), anti-CBP (ThermoFisher, PA1–847), anti-FOXA1 (ThermoFisher, PA5–27157), and anti-β-actin (Santa Cruz, sc-130,656).

### ChIP-seq and GRO-seq data analyses

ChIP-seq data analyses were used to search for putative enhancers of *CYR61*, and GRO-seq data analysis was used to detect expression of eRNAs at putative enhancer regions. ChIP-seq data of colon adenocarcinoma samples and normal colon tissues were downloaded from the GEO database, Data for ChIP-seq and GRO-seq were downloaded from the GEO database and listed in Additional file 1: Table S2. Raw data were aligned using Bowtie2 [27] (version 2.2.9) to the human genome (build hg38, GRCh38) with parameter -p 15 -x after quality filtering. MACS [28] (version 1.4.2) was used with the parameter “-f BAM -g hs -n sample -p 1e-5 -B” for peak calling. The ChIP-seq and GRO-seq figures were visualized in the UCSC Genome Brower.

### Chromatin immunoprecipitation (ChIP)

ChIP-qPCR was used to detect the histone modification and TF binding at the promoter and enhancer regions of *CYR61*. As we described before [29], the ChIP assay was performed using a Magna ChIP™ G Chromatin Immunoprecipitation Kit (Millipore, 17–611) according to the manufacturer’s protocol. ChIP-grade antibodies were as follows: 2 μg anti-RNA polymerase II (Millipore, 05–623), 2 μg anti-H3K27ac (ThermoFisher, 720,096), anti-H3K4me1 (Abcam, ab8895), 2 μg anti-H3K4me3 (Millipore, 17–614), 3 μg anti-CBP (ThermoFisher, PA1–847), and 3 μg anti-FOXA1 (ThermoFisher, PA5–27157). Immunoprecipitated DNA was detected by qPCR and normalized with input DNA. The sequences of the primers are listed in Additional file 1: Table S3.

### RNA interference (RNAi) assay

All siRNAs were designed by GenePharma (Shanghai, China). Cells were transfected with siRNA using Lipofectamine RNAiMAX Reagent (Invitrogen, 13,778,100) according to the manufacturer’s protocol. The sequences of the siRNAs are listed in Additional file 1: Table S4.

### Dual-luciferase reporter assay

Dual-luciferase reporter assays were used to detect putative enhancer activity. The pGL3-basic Vector (Promega, E1751) and pRL-SV40 Vector (Promega, E2231) were purchased. The pGL3-basic-promoter plasmid was constructed by inserting the *CYR61* promoter region into the pGL3-basic plasmid, and the putative enhancer fragment or negative control region was inserted into the pGL3-basic-promoter plasmid to construct the pGL3-basic-promoter-enhancer/NC plasmids. Primers and insertion sites for dual-luciferase reporter assays are shown in Additional file 1: Table S5. All constructed plasmids were verified by sequencing. Plasmid transfection was performed with FuGENE HD transfection reagent (Promega, E2312) according to the manufacturer’s protocol. Forty-eight to seventy-two hours after transfection, cells were harvested. Luciferase activity was determined by the dual-luciferase reporter assay system (Promega, E1910).

### Chromosome conformation capture (3C) assay

Chromosome conformation capture (3C) assay was used to detect physical interactions between enhancer and promoter [30, 31]. The 3C assay was performed as described previously [32, 33]. HindIII was used for genomic DNA digestion. Plasmids used in the 3C assays were BAC clones for *ERCC3* (Invitrogen, CTD-3251 N23) and *CYR61* (Invitrogen, RP11-963G4). Primers used in this assay are shown in Additional file 1: Table S6.

### Transwell assay

Cell migration assays were performed in a transwell chamber (24-well, 8 μm pore size; Corning). Plasmids GV219-mCYR61 and GV219-mFOXA1 (GeneChem, China) were used to over-express CYR61 and FOXA1. Cells were transfected with siRNA at 25 pmol and/or GV219 plasmid, 2.8 μg each, in a 6-well plate for 48~72 h, and then 1.0 × 10^5^ cells were transplanted into a transwell chamber. After 48 h, migrated cells were stained with crystal violet. Five random fields at 200× magnification were used for cell counting for each membrane.

### FOXA1 overexpression and *CYR61* shRNA lentivirus vectors transduction

Lentiviral vector GV344 (LV-firefly_Luciferase-puromycin/shCYR61) was used to knockdown CYR61, and lentiviral vector CV572 (LV-Cherry-neomycin/FOXA1) was used to overexpress FOXA1. All lentiviral vectors were constructed by the Shanghai GenePharma Corporation (Shanghai, China). The targeting sequence of shCYR61 was 5′- GCATCCTATACAACCCTTT − 3′. A six-well plate was inoculated with 2 × 10^5^ HCT116 cells. Twenty-two hours after inoculation, LV-firefly_Luciferase-puromycin/shNC and LV-firefly_Luciferase-puromycin/shCYR61 lentivirus were used to infect cultured cells. Fourteen hours later, the medium in each well was changed with fresh normal medium. Seventy-two hours after infection, 2 μg/ml puromycin (Sangon Biotech, China, # A610593) was used to screen stable cell clones. The transduction of FOXA1 overexpression lentivirus was performed after puromycin screening for 7 days and the transduction procedure was as shCYR61‘s. Seventy-two hours after infection with LV-Cherry-neomycin/con and LV-Cherry-neomycin/FOXA1, 800 μg/ml G418 (Sangon Biotech, China, # B540723) was used to select stable cell clones.

### Animal studies

Six-week old male NOD SCID mice (Beijing Vital River Laboratory Animal Technology Co., Ltd., China, #406) weighing between 19 g to 22 g were used in our animal study. HCT116 cells (3 × 10^6^ cells) transduced with lentivirus were tail vein injected into the mice (*n* = 5 per group). Tumor metastasis were monitored weekly by an in vivo imaging system (Perkin Elmer IVIS Lumina III) from the third week after injection. Mice were intraperitoneally injected with 150 μg D-luciferin (Solarbio, China, #D8390) solution per gram body weight for 10 min before subjected to bioluminescent imaging. Tumor size and metastasis were quantified using Living Image software (Perkin Elmer IVIS Lumina III).

### Statistical analysis

Statistical analyses were conducted in SPSS Statistics 19.0. All data shown were determined for three independent experiments unless otherwise stated, and presented as the mean ± S.D., *P < 0.05, **P < 0.01, ***P < 0.001.

## Results

### CYR61 is elevated in primary colonic adenocarcinoma tissue and colon cancer cell lines

**Fig. 1:**
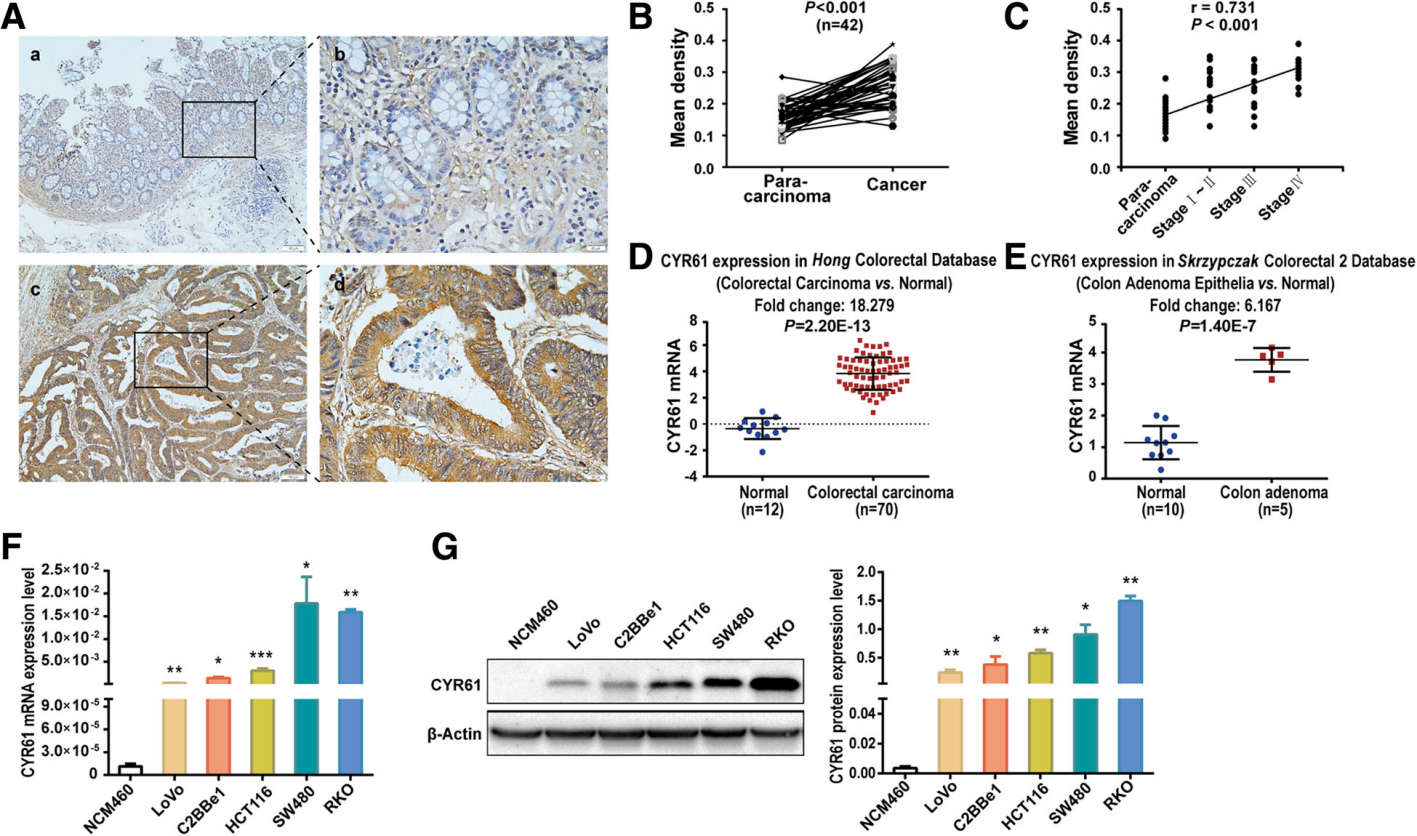
*CYR61* expression levels in primary colonic adenocarcinoma patient samples and colon cell lines. **a** Immunostaining for CYR61 in paraffin sections of para-carcinoma tissues (*a*, *b* and cancer tissues (*c*, *d* from patients with colon adenocarcinoma (magnification, × 100, × 400). **b** The mean density of CYR61 in IHC of colon para-carcinoma and cancer tissues from 42 cases; significance determined by the paired-samples t-test. **c** Correlation between the mean density of CYR61 in IHC and TNM levels, significance determined by Spearman’s rank correlation. **d** CYR61 mRNA expression levels in primary colon cancer tissues compared with normal controls by analysis of the Hong Colorectal microarray dataset. **e** CYR61 mRNA expression levels in primary colon cancer tissues compared with normal controls by analysis of the Skrzypczak Colorectal 2 microarray dataset. **f** Expression of CYR61 mRNA and **g** protein levels in human colon cell lines, significance determined by the independent samples t-test. Data are shown as mean ± S.D., *n* = 3. *P < 0.05, **P < 0.01, ***P < 0.001

We initially performed immunohistochemical analysis (IHC) to examine CYR61 protein expression in specimens from 42 cases of colonic adenocarcinomas and matched para-carcinoma tissues. The results showed that the expression levels of CYR61 protein in carcinoma tissue were significantly higher than those in para-carcinoma tissue (Fig. 1a, b). A significant association was noted between CYR61 protein expression and histological tumor grade (*p* < 0.001) (Fig. 1c). No significant correlation was seen between CYR61 expression and other clinicopathological parameters examined (Additional file 1: Figure S1, Table S7). ONCOMINE microarray dataset analysis from the Hong Colorectal dataset and Skrzypczak Colorectal 2 dataset showed that *CYR61* mRNA was significantly up-regulated in primary colon adenocarcinoma tissues compared with normal controls (Fig. 1d, e), consistent with our tissue specimen analysis, indicating that *CYR61* expression is up-regulated in primary colonic adenocarcinoma tissue. Furthermore, higher mRNA and protein expression of *CYR61* was observed in several colon cancer cell lines (LoVo, C2BBe1, HCT116, SW480, and RKO), compared with the normal human colon mucosal epithelial cell line NCM460 (Fig. 1f, g). Thus, these results indicate that high expression of *CYR61* is associated with tumor development and aggressiveness in colonic adenocarcinoma tissue and cell lines.

### *CYR61* expression is regulated by enhancers in colon cancer

**Fig. 2:**
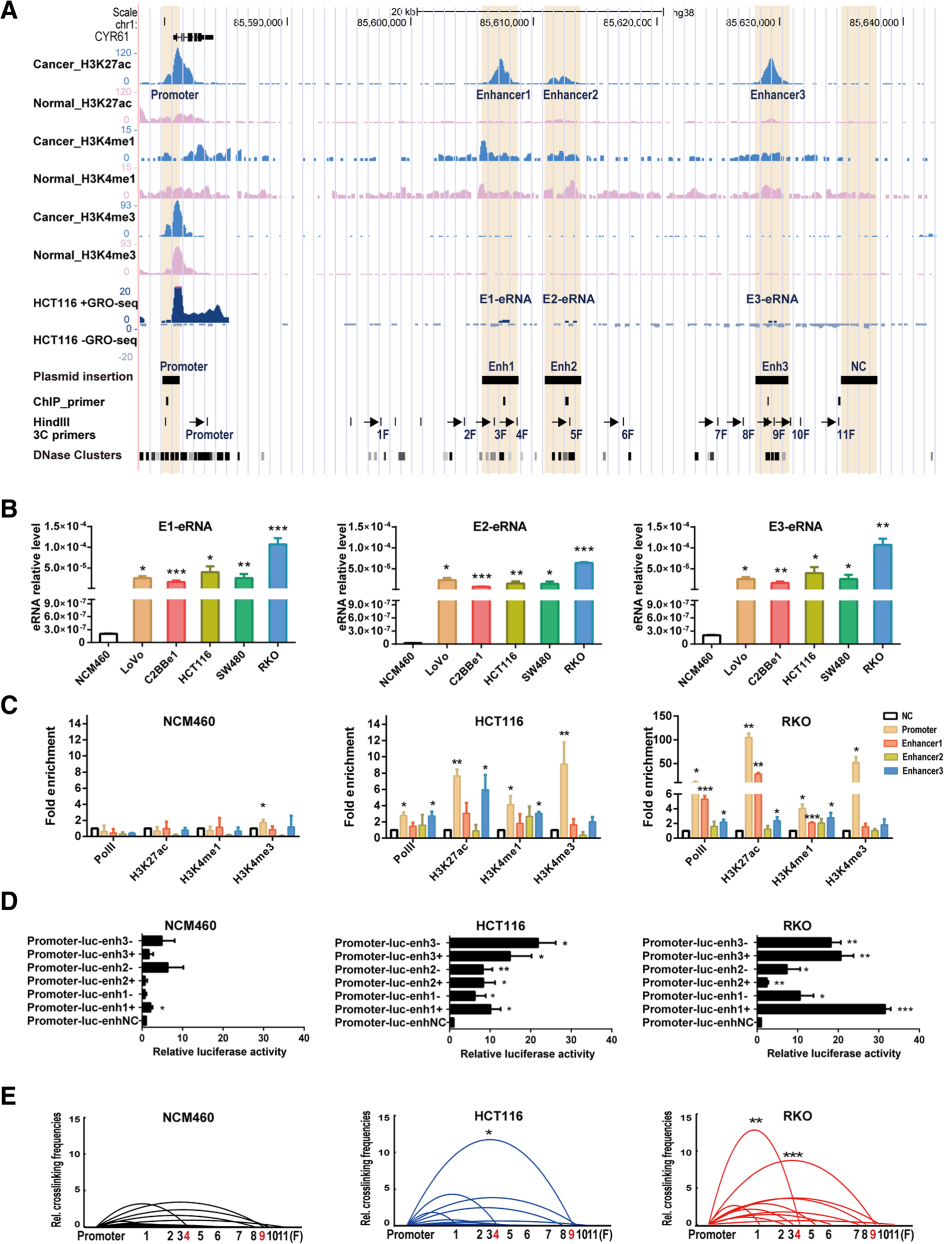
Identification of CYR61 enhancers. **a** From top to bottom: UCSC gene annotation (GRCh38/ hg38) of CYR61; enrichment of H3K27ac in colon adenocarcinoma and normal colon mucosa; enrichment of H3K4me1 in colon adenocarcinoma and normal colon mucosa; enrichment of H3K4me3 in colon adenocarcinoma and normal colon mucosa; GRO-seq of eRNA expression in HCT116 cells; locations of fragments inserted in the pGL3-basic plasmid; positions of primers used in ChIP-qPCR; HindIII digestion sites, and positions of primers used in the 3C assay; DNase I hypersensitivity signals. NC: negative control sequence. **b** Expression levels of eRNA in different colon cell lines. **c** RNA polymerase II, H3K27ac, H3K4me1 and H3K4me3 enrichment in NCM460, HCT116 and RKO cells, as assessed by ChIP-qPCR and expressed as fold change over input normalized to the NC. **d** Relative enhancer luciferase activities, normalized to expression of Renilla luciferase from a co-transfected pRL-SV40 plasmid. **e** Relative cross-linking frequencies between the constant region (CYR61 promoter) and distal fragments (F1~F11) in the three cell lines, measured by qPCR, normalized to *ERCC3* and compared to the control region (fragment F1) to calculate the relative fold change. Significance for all data except **a** determined by the independent sample t-test, and data are shown as mean ± S.D., *n* = 3. *P < 0.05, **P < 0.01, ***P < 0.001

Several recent studies have shown that enhancers promote gene transcription via a long-range interaction with their cognate promoters and distinct histone modifications in surrounding nucleosomes [16, 34], so we determined whether there were putative enhancers that interacted with the *CYR61* promoter and promoted *CYR61* expression in colon cancer cell lines. Firstly, we analyzed ChIP-seq data, of colon adenocarcinoma samples and normal colon tissues, downloaded from the GEO database and found three putative enhancers located at 26.5 kb, 31.5 kb, 48.6 kb downstream of the *CYR61* transcriptional start site (Fig. 2a), referred to as Enhancer1 (chr1:85605899–85,608,570), Enhancer2 (chr1: 85610948–85,613,635) and Enhancer3 (chr1: 85628077–85,630,633), respectively. GRO-seq data of HCT116 showed all three regions transcribed eRNAs, denoted E1-eRNA, E2-eRNA, and E3-eRNA. These features are consistent with characteristics of an activated enhancer [17].

We detected eRNA expression at the three putative enhancer regions in NCM460 and colon cancer cell lines. In agreement with the results of the GRO-seq analysis, real-time quantitative PCR analysis showed that colon cancer cells expressed dramatically higher eRNA levels than NCM460 (Fig. 2b).

To investigate *CYR61* enhancer activity in the above cell lines, we detected histone modification and RNA polymerase II (Pol II) enrichment in NCM460, HCT116 and RKO cell lines by ChIP-qPCR. As shown in Fig. 2c, H3K27ac, H3K4me1 and RNA polymerase II were not enriched at the three putative enhancer regions in NCM460 cells, but were enriched at Enhancer3 in HCT116 cells. H3K27ac ChIP-seq data, downloaded from GEO, showed high H3K27ac enrichment at the Enhancer3 region in HCT116 cells, but low H3K27ac enrichment at Enhancer1 and Enhancer2 regions (Additional file 1: Figure S2A), consistent with our ChIP-qPCR assay. In RKO cells, the H3K27ac and Pol II enrichment was observed at both Enhancer1 and Enhancer3 regions. Next, a dual-luciferase reporter assay was used to further determine the enhancer activity. The fragments inserted into the plasmid are shown in Fig. 2a. The inserted *CYR61* promoter showed significantly higher promoter activity in HCT116 and RKO cells than that in NCM460 cells (Additional file 1: Figure S2B). Furthermore, all enhancers, inserted in both forward and reverse directions, showed enhancer activity in the above colon cancer cell lines (Fig. 2d).

As mentioned above, typically, enhancers contact with the promoters of their cognate gene through long-range interactions. We next determined whether the putative enhancers interacted with the *CYR61* promoter by using a chromatin conformation capture (3C) assay. As shown in Fig. 2e, a strong interaction between Enhancer3 and the CYR61 promoter was identified in colon cancer cells HCT116 and RKO. The ninth test fragment (9F in Fig. 2a), which overlaps with the Enhancer3 region, displayed significantly higher interaction frequency with the CYR61 promoter compared to the neighboring DNA fragments (1F in Fig. 2a). Therefore, the 2.5 kb region located 48.6 kb away from the CYR6 promoter has all characteristics of an active enhancer (high level of H3K27ac and H3K4me1, transcription enhancement, and interaction with the *CYR61* promoter). Interestingly, the interaction between Enhancer1 and the *CYR61* promoter occurred at high frequency in RKO cells, but not in HCT116 cells. In NCM460 cells, no interaction between these putative enhancers and the *CYR61* promoter was observed. These results imply that colon cancer cells exhibit different CYR61 regulation patterns by different enhancers. Moreover, the differential activity of enhancers between these cells further underscores the concept that these enhancers are only activated in cancer cells and promoted *CYR61* transcription.

### FOXA1 regulates enhancer activity to promote *CYR61* expression

**Fig. 3:**
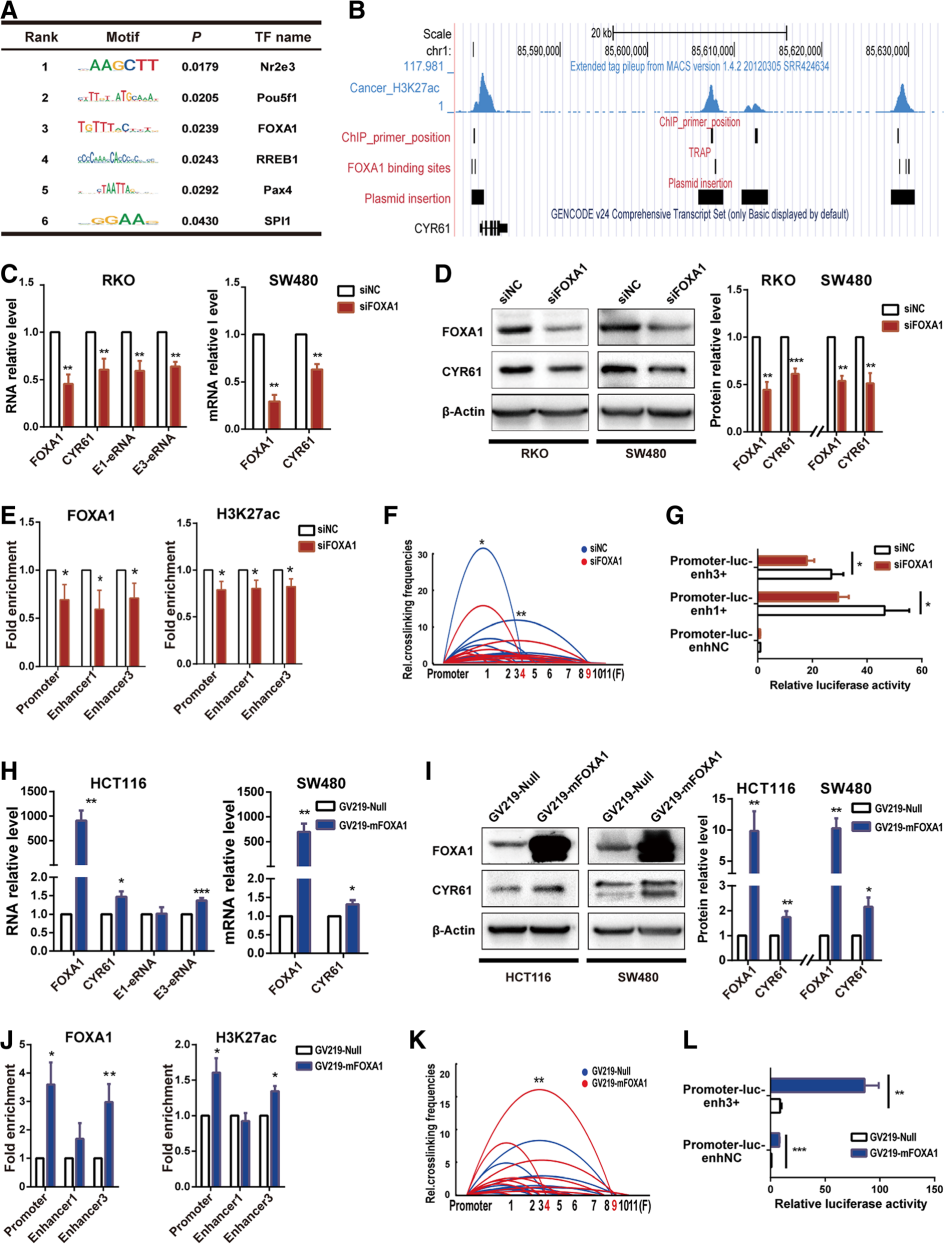
FOXA1 regulates enhancer activity to promote CYR61 expression. **a** TF binding to the CYR61 promoter, Enhancer1 and Enhancer3 were predicted by TRAP. **b** FOXA1 binding site predicted by TRAP. **c** CYR61 mRNA and eRNA levels decreased after treatment with FOXA1 siRNA for 72 h in RKO and SW480 cell lines. **d** CYR61 protein levels decreased after treatment with FOXA1 siRNA for 72 h in RKO and SW480 cell lines. **e** FOXA1 and H3K27ac enrichment after treatment with FOXA1 siRNA for 72 h in the RKO cell line, as assessed by ChIP-qPCR. **f** Relative crosslinking frequencies of CYR61 promoter/Enhancer1 and CYR61 promoter/Enhancer3 decreased after treatment with FOXA1 siRNA for 72 h in RKO cells, as assessed by 3C; significance determined by the paired-samples t-test. **g** Relative activity of promoter and enhancers after treatment of RKO cells with FOXA1 siRNA for 72 h, as assessed by relative luciferase reporter gene activity. (H) CYR61 mRNA and eRNA levels increased after transfection with GV219-mFOXA1 for 48 h in HCT116 and 72 h in SW480 cell lines. **i** CYR61 protein levels increased after transfection with GV219-mFOXA1 for 48 h in HCT116 and 72 h in SW480 cells. **j** FOXA1 and H3K27ac enrichment after transfection with GV219-mFOXA1 for 48 h in HCT116 cells, as assessed by ChIP-qPCR. **k** Relative crosslinking frequencies of CYR61 promoter/Enhancer3 were increased after transfection with GV219-mFOXA1 for 48 h in HCT116 cells, as assessed by 3C; significance determined by the paired-samples t-test. **l** Relative activity of promoter and enhancers after transfection of HCT116 cells with GV219-mFOXA1 for 48 h, as assessed by relative luciferase reporter gene activity. Significance for all data except **a**, **b**, **f** and **k** was determined by the independent samples t-test. Data are shown as mean ± S.D., *n* = 3. *P < 0.05, **P < 0.01, ***P < 0.001

Most enhancer activators function by binding sequence-specifically to enhancers and promoters and forming protein-protein interactions with RNA polymerase and other general TFs. So, we determined the sequence-dependent TFs that mediate *CYR61* promoter and enhancer interaction. By analyzing the *CYR61* promoter sequence (chr1:85579761–85,580,961), Enhancer1 sequence (chr1:85605899–85,608,570) and Enhancer3 sequence (chr1:85628077–85,630,633) using transcription factor affinity prediction (TRAP) web tools (http://trap.molgen.mpg.de/cgi-bin/trap_multi_seq_form.cgi) [35], we found 6 TFs with statistically significant *p*-values (Fig. 3a). FOXA1, a pioneer factor in many physiological and pathological conditions was among the TFs listed and showed enrichment at TRAP-predicted loci (Fig. 3b). Furthermore, pioneer factors can bind to compacted chromatin to facilitate the binding of other TFs [22, 23]. Based on this, we reasoned that FOXA1 may regulate *CYR61* enhancer activity and promote *CYR61* expression.

We determined the enrichment of FOXA1 at the TRAP-predicted loci. FOXA1 was not enriched at TRAP-predicted loci in NCM460 cells, but enriched at the *CYR61* promoter, Enhancer1 and Enhancer3 loci in RKO cells, and enriched at the *CYR61* promoter and Enhancer3 loci in HCT116 cells (Additional file 1: Figure S3A-C). We next determined whether knockdown of FOXA1 could decrease enhancer activity and lower *CYR61* expression. Supporting a role for FOXA1 in enhancing *CYR61* expression, knockdown of FOXA1 expression with FOXA1-specific siRNA markedly reduced *CYR61* expression both at the mRNA and protein levels in RKO and SW480 cells, and the eRNA levels were also reduced in RKO cells as well (Fig. 3c, d). Moreover, ChIP-qPCR results showed that FOXA1 knockdown reduced FOXA1 enrichment at both Enhancer1 and Enhancer3 loci, and reduced H3K27ac enrichment at the target loci (Fig. 3e). In parallel with the decrease of FOXA1 occupancy at the *CYR61* promoter, Enhancer1 and Enhancer3 loci, and the *CYR61* promoter/Enhancer1 and *CYR61* promoter/Enhancer3 looping frequencies were decreased (Fig. 3f). To distinguish the contribution of FOXA1 to the individual *CYR61* promoter, Enhancer1, and Enhancer3 activities, we knocked down FOXA1 by siRNA and performed a dual-luciferase assay. Both promoter-luc-Enhancer1+ and promoter-luc-Enhancer3+ showed decreased activity after FOXA1 knockdown, but the activity of promoter-luc-enhNC plasmid (which contained an NC fragment instead of an enhancer fragment) tended to be slightly decreased, but was not statistically different from the negative control group (Fig. 3g).

To further confirm the above results, we over-expressed FOXA1 by transfecting a GV219-mFOXA1 plasmid in HCT116 and SW480 cells, then detected *CYR61* expression. Increasing FOXA1 expression markedly increased *CYR61* expression at both the mRNA and protein levels, and this paralleled an increase in eRNA levels (Fig. 3h, i). In addition, FOXA1 over-expression increased both FOXA1 enrichment and H3K27ac enrichment at the promoter and Enhancer3 loci in HCT116 cells (Fig. 3j), and the *CYR61* promoter/Enhancer3 looping frequencies were increased by FOXA1 over-expression (Fig. 3k). The dual-luciferase assay showed both promoter-luc-enhNC and promoter-luc-Enh3+ plasmid activities were increased after FOXA1 over-expression, with promoter-luc-Enh3+ showing a greater increase than promoter-luc-enhNC (Fig. 3l). Together, these results demonstrate that FOXA1 regulates *CYR61* expression mainly by regulating its enhancer activity.

### CBP collaborates with FOXA1 to regulate *CYR61* enhancer activity and *CYR61* expression

**Fig. 4:**
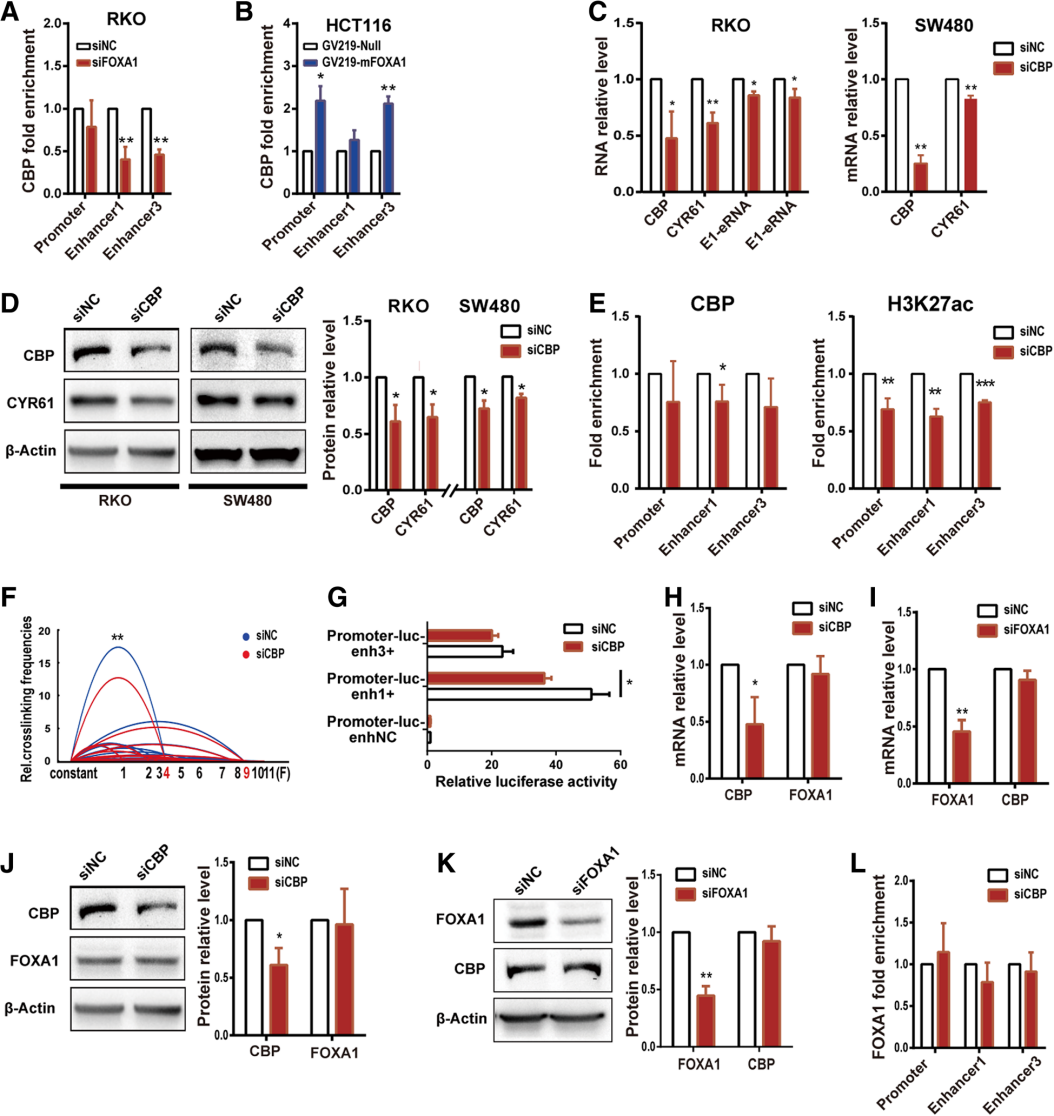
CBP is recruited by FOXA1 to up-regulate CYR61 enhancer activity and CYR61 expression. **a** CBP enrichment decreased at Enhancer1 and Enhancer3 after treatment with FOXA1 siRNA for 72 h in RKO cells, and **b** increased at the promoter and Enhancer3 after FOXA1 over-expression for 48 h in HCT116 cells, as assessed by ChIP-qPCR. **c** CYR61 mRNA and eRNA, and **d** protein levels were decreased after treatment with CBP siRNA for 26 h in RKO and SW480 cell lines. **e** CBP and H3K27ac enrichment was decreased after treatment with CBP siRNA for 26 h in RKO cells, as assessed by ChIP-qPCR. **f** Relative crosslinking frequencies of CYR61 promoter/Enhancer1 were decreased after treatment with CBP siRNA for 26 h in RKO cells, as assessed by 3C; significance determined by the paired-samples t-test. **g** Relative activity of promoter and enhancers after treatment with CBP siRNA for 26 h in RKO cells, as assessed by relative luciferase reporter gene expression. CBP and FOXA1 mRNA expression levels in RKO cells after treatment with CBP siRNA for 26 h **h** or with FOXA1 siRNA for 72 h **i**. CBP and FOXA1 protein expression in RKO cells after treatment with CBP siRNA for 26 h **j** or with FOXA1 siRNA for 72 h **k**. (L) FOXA1 enrichment after treatment with CBP siRNA for 26 h in RKO cells, as assessed by ChIP-qPCR. Significance for all data except **f** was determined by the independent samples t-test. Data are shown as mean ± S.D., *n* = 3. *P < 0.05, **P < 0.01, ***P < 0.001

In the above experiments, we also found that with up- or down-regulation of FOXA1, the enrichment of CREB-binding protein (CBP) at Enhancer1, Enhancer3 and promoter loci changed accordingly (Figs. 3e, j, 4a, b, Additional file 1: Figure S3D-F). To validate an involvement of the histone acetyltransferase CBP in the regulation of enhancer activity, we knocked down CBP by siRNA and detected both *CYR61* expression and enhancer activity. The results showed that, following CBP knockdown, both mRNA and protein levels of *CYR61* were decreased in RKO and SW480 cells (Fig. 4c, d), and the expressions of E1-eRNA and E3-eRNA were likewise decreased in RKO cells (Fig. 4c). ChIP-qPCR analysis showed that, after CBP knockdown, CBP enrichment was statistically reduced at the Enhancer1 locus, and caused a decrease of H3K27ac enrichment at the *CYR61* promoter, Enhancer1 and Enhancer3 loci (Fig. 4e). Parallel to the decreased CBP enrichment, *CYR61* promoter/Enhancer1 looping frequencies and promoter-luc-Enhancer1+ plasmid activity were decreased after CBP knockdown (Fig. 4f, g). These data indicate that CBP is required for the maintenance of H3K27Ac histone modification and promoter-enhancer 3D structure. Additionally, CBP and FOXA1 knockdown did not affect each other’s expression level (Fig. 4h-k). Interestingly, FOXA1 knockdown decreased the CBP enrichment at the target loci (Fig. 4a), while CBP knockdown did not affect FOXA1 enrichment (Fig. 4l), suggesting that enrichment of FOXA1 is necessary for the recruitment of CBP at target loci.

### Small molecule compounds regulate *CYR61* expression through affecting enhancer activity

**Fig. 5:**
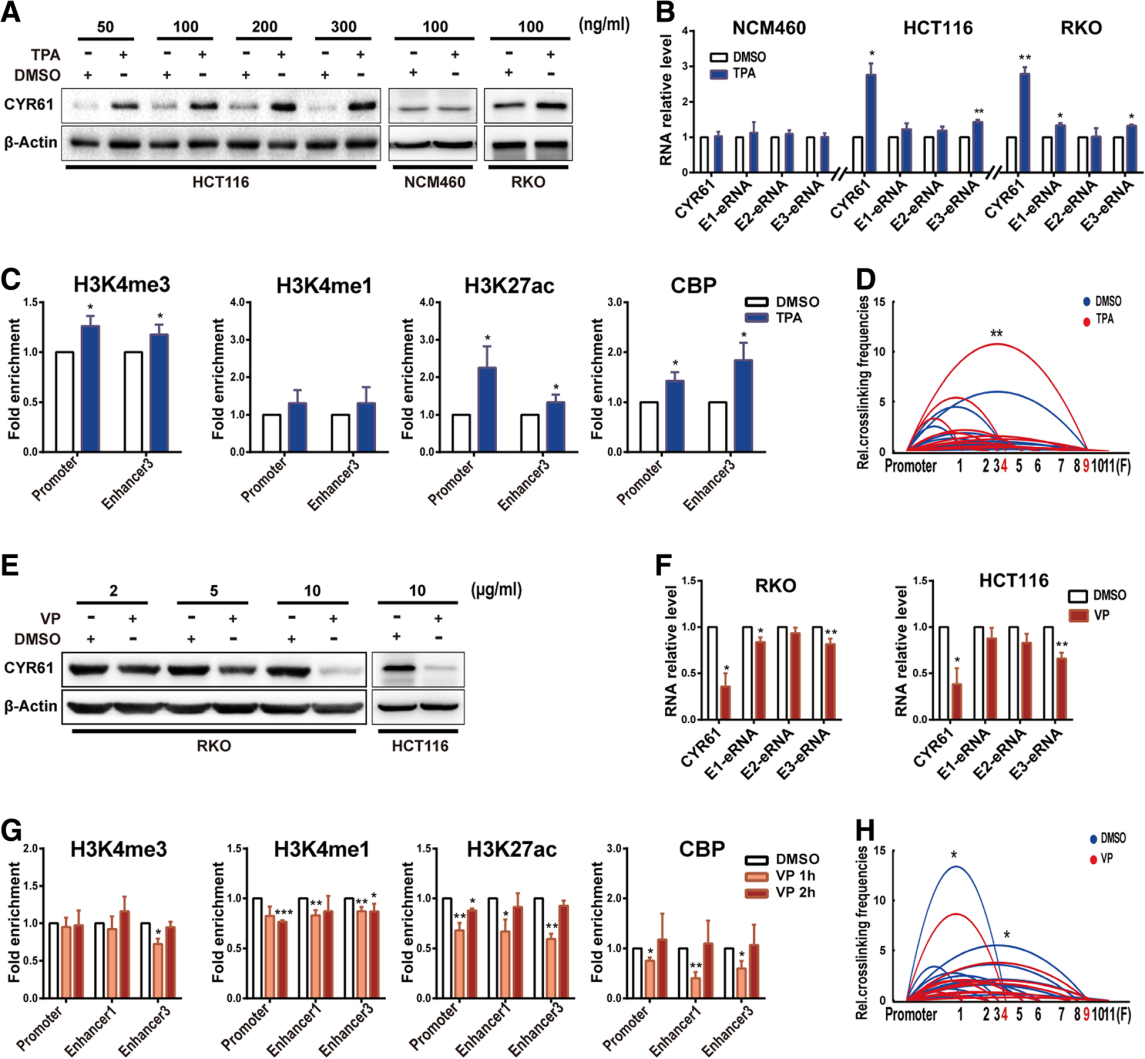
Small molecule compounds regulate CYR61 expression through affecting enhancer activity. **a**-**d** TPA treatment. **a** CYR61 protein, and **b** CYR61 mRNA and eRNA levels after treatment of NCM460, HCT116, and RKO cells with TPA for 2 h. (C) H3K4me3, H3K4me1, H3K27ac, and CBP enrichment after treatment with 100 ng/ml TPA for 2 h in HCT116 cells, as assessed by ChIP-qPCR. **d** Relative crosslinking frequencies of CYR61 promoter/Enhancer3 were increased after treatment with 100 ng/ml of TPA for 2 h in HCT116 cells, as assessed by 3C, significance determined by paired-samples t-test. **e**, **h** VP treatment. **e** CYR61 protein, and **f** CYR61 mRNA and eRNA levels after treatment of RKO and HCT116 cells with VP for 2 h. **g** H3K4me3, H3K4me1, H3K27ac and CBP enrichment after treatment with 10 μg/ml VP for 1 or 2 h in RKO cells, as assessed by ChIP-qPCR. **h** Relative crosslinking frequencies of the CYR61 promoter/Enhancer1 and CYR61 promoter/Enhancer3 were decreased after treatment of RKO cells with 10 μg/ml VP for 1 h, as assessed by 3C; significance determined by the paired-samples t-test. Significance for all data except **d** and **h** was determined by the independent samples t-test. Data are shown as mean ± S.D., n = 3. *P < 0.05, **P < 0.01, ***P < 0.001

12-O-tetradecanoyl phorbol-13-acetate (TPA) is reported to up-regulate expression of *CYR61* in breast cancer cells [36]. Here, TPA treatment promoted the expression of *CYR61* in both HCT116 and RKO cells (Fig. 5a, b, Additional file 1: Figure S4A-C), but not in NCM460 cells. To investigate whether the enhancers were affected, we detected eRNA expression and found that E3-eRNA in HCT116 cells, and both E1-eRNA and E3-eRNA in RKO cells were elevated by 100 ng/ml TPA treatment for 2 h (Fig. 5b). Further, our ChIP-qPCR results showed that TPA treatment caused H3K27ac, H3K4me3 and CBP enrichment at the *CYR61* promoter and Enhancer3 region in HCT116 cells (Fig. 5c), indicating that TPA treatment induced enhancer activity. In addition, the above findings were confirmed by 3C analysis showing that *CYR61* promoter/Enhancer3 looping frequencies were statistically increased in HCT116 after TPA treatment (Fig. 5d).

In addition to TPA, we characterized the effects of another small molecule drug, verteporfin (VP), a benzoporphyrin derivative, which has been reported to inhibit cell growth in cancer cells through down-regulating *CYR61* expression [37, 38]. Therefore, we tested whether VP treatment could inhibit expression of *CYR61* and suppress enhancer activity in colon cancer cells. VP treatment inhibited the expression of *CYR61* mRNA and protein in both RKO and HCT116 cells, E3-eRNA in HCT116 cells, and both E1-eRNA and E3-eRNA in RKO cells (Fig. 5e, f, Additional file 1: Figure S4D-F). Since *CYR61* mRNA levels were decreased in a time-dependent manner after treatment with 10 μg/ml VP for various times and bottomed out at 2 h (Additional file 1: Figure S4 E), we inferred that H3K4me1, H3K27ac, and CBP enrichment would bottomed out at 2 h also, but in fact, only H3K4me1 and H3K27ac enrichment at CYR61 promoter region showed significant decreases after VP treatment for 2 h. Considering that TF enrichment and histone modifications may change earlier than the target gene does, we tested the TF enrichment and histone modifications after VP treatment for 1 h, and found that treatment with VP for 1 h significantly inhibited H3K4me1, H3K27ac and CBP enrichment at the *CYR61* promoter, Enhancer1 and Enhancer3 regions in RKO cells (Fig. 5g). These results suggested that the CBP enrichment and active histone modifications changed earlier than the target gene. CY*R61* promoter/Enhancer1 and *CYR61* promoter/Enhancer3 looping frequencies were statistically reduced after treatment of RKO cells with VP, as shown by 3C assay (Fig. 5h). These data suggest that TPA and VP regulate *CYR61* expression at least partially through regulating *CYR61* enhancer activity.

### FOXA1 promotes colon cancer cell metastasis by increasing *CYR61* enhancer activity

**Fig. 6:**
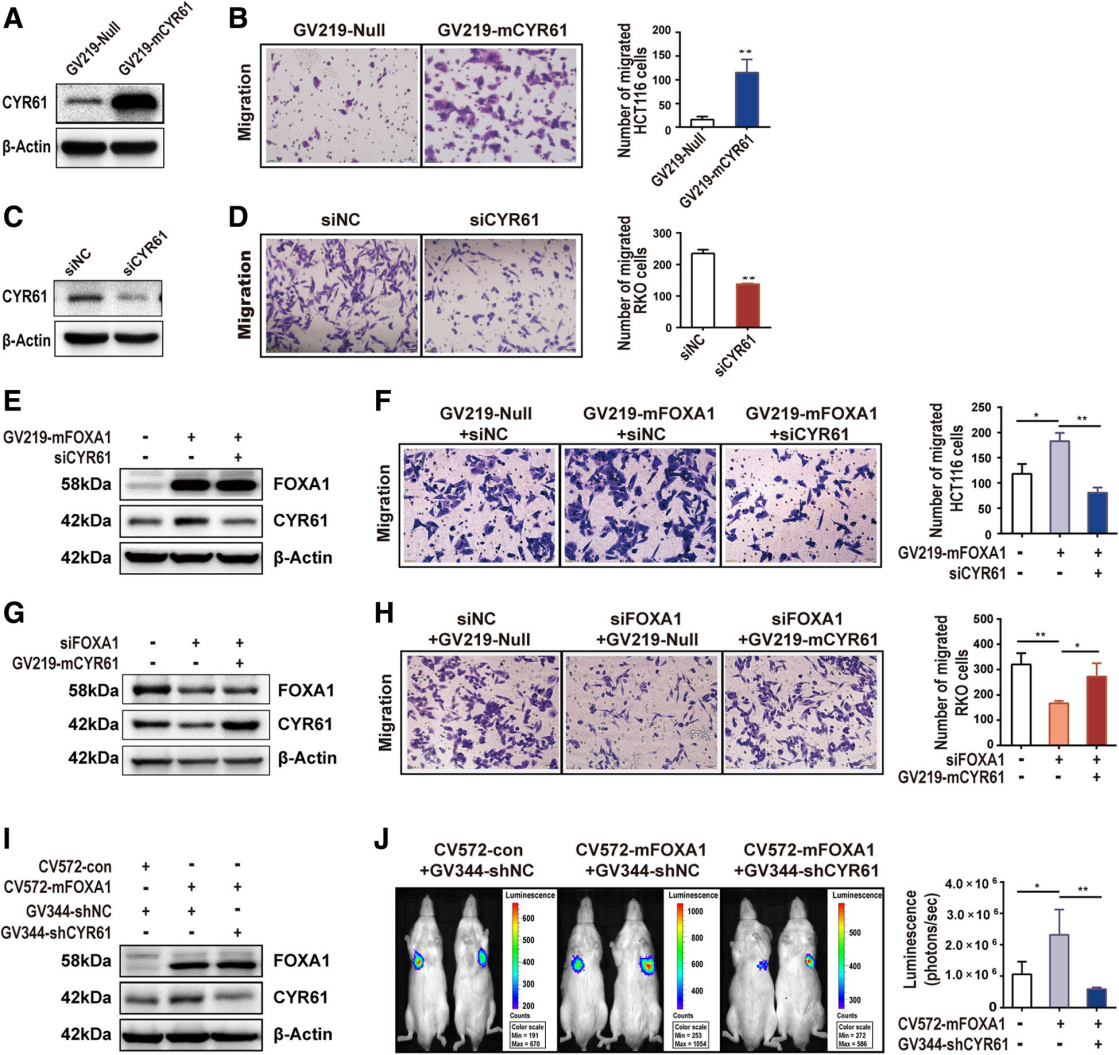
FOXA1 promotes colon cancer cell migration by increasing enhancer activity of *CYR61*. **a** CYR61 protein levels were increased 48 h after transfection with GV219-mCYR61 in HCT116 cells. **b** CYR61 ectopic expression increased HCT116 cell migration. **c** CYR61 protein levels were decreased after transfection with CYR61 siRNA for 48 h in RKO cells. **d** Knockdown of CYR61 decreased RKO cell migration. **e** CYR61 protein levels were increased after ectopic expression FOXA1 for 48 h and restored by transfection of HCT116 cells with CYR61 siRNA for 48 h. **f** FOXA1 ectopic expression increased HCT116 cell migration, while CYR61 knockdown restored HCT116 cells migration. **g** CYR61 protein levels were decreased after transfection with FOXA1 siRNA for 72 h and restored by ectopic expression of CYR61 for 48 h in RKO cells. **h** FOXA1 Knockdown decreased RKO cell migration, while ectopic expression of CYR61 partially restored RKO cell migration. **i** CYR61 protein levels were increased after LV-Cherry-neomycin/FOXA1 lentivirus (CV572-mFOXA1) transduction and restored by transduction of HCT116 cells with CYR61 shRNA (GV344-shCYR61). **j** Representative images of metastatic signal detected by IVIS in NOD SCID mice 28 days after tail vein injection (*n* = 5/group). Quantification of the luciferase signal by IVIS. Significance for all data was determined by the independent samples t-test. Data are shown as mean ± S.D., *n* = 3. *P < 0.05, **P < 0.01

**Fig. 7:**
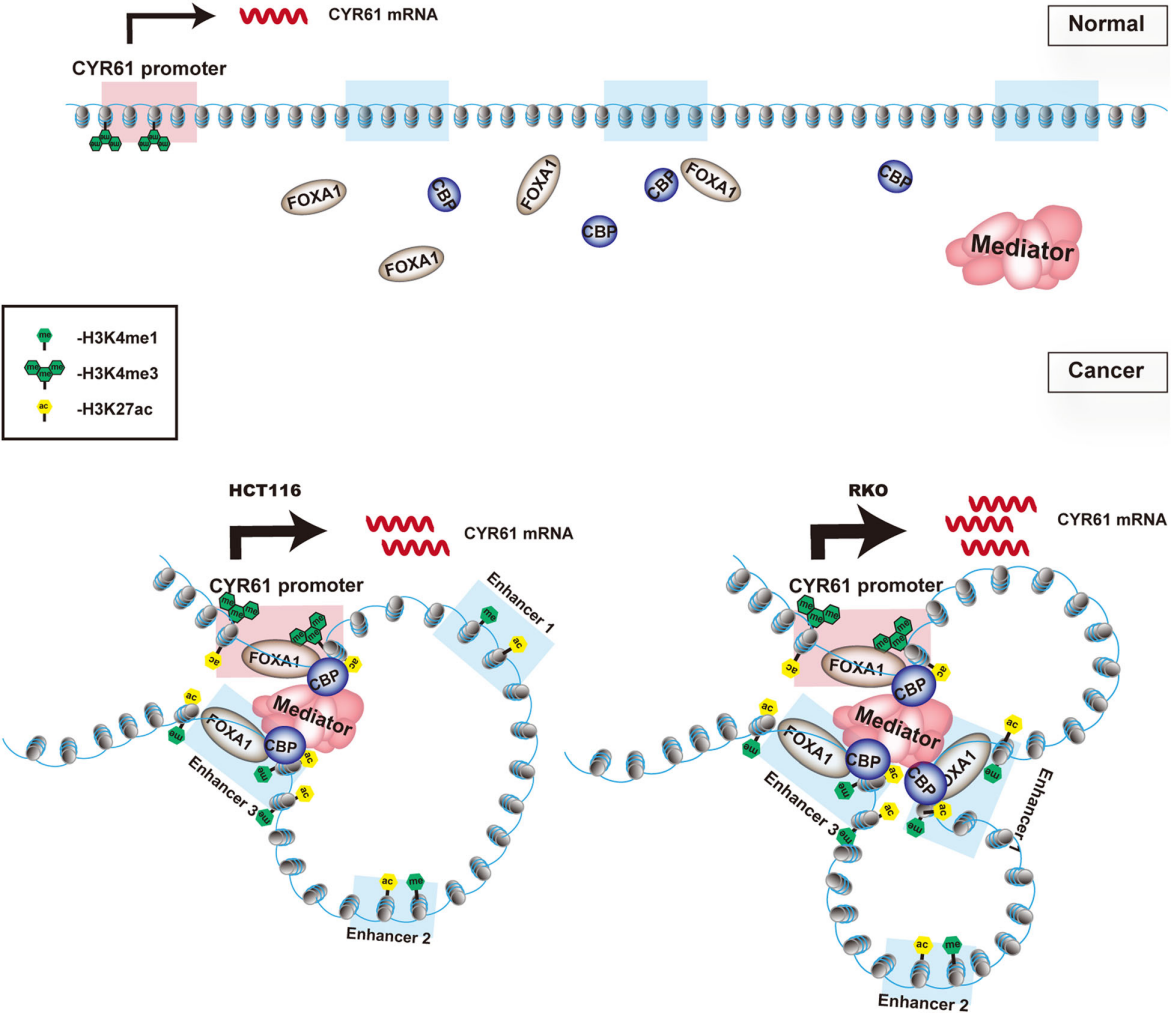
Schematic representation of enhancer regulation of CYR61 expression. Under normal conditions, CYR61 mRNA is transcribed at a low level. In colon cancer, FOXA1 and CBP mediate aberrant promoter-enhancer looping and help to establish the active chromatin state, which leads to an increase of CYR61 transcription

Since CYR61 is a key regulator of migration [7, 8, 39, 40], a transwell assay was performed to explore the functional roles of the enhancers in cancer progression. Ectopic expression of CYR61 increased the migration of HCT116 cells (Figs. 6a, 7b). Conversely, knockdown of CYR61 decreased the migration of RKO cells (Fig. 6c, d). Ectopic FOXA1 expression up-regulated CYR61 expression and increased HCT116 cell migration. Conversely, CYR61 knockdown, in cells ectopically expressing FOXA1, decreased cell migration (compared with ectopic FOXA1 expressing cells) (Fig. 6e, f). Accordingly, FOXA1 knockdown down-regulated CYR61 expression and decreased RKO cell migration, whereas ectopic expression of CYR61 in FOXA1-knockdown cells increased cell migration (Fig. 6g, h).

To verify whether FOXA1 could promote colon cancer cells metastasis by up-regulating CYR61 expression in vivo, we constructed mouse models by tail vein injection of cancer cells and observing lung metastases. We found that over-expression of FOXA1 up-regulated CYR61 expression levels and increased the metastatic potential of HCT116 cells; Conversely, CYR61 knockdown, in cells ectopically expressing FOXA1, decreased cell metastasis (Fig. 6i, j). The above data suggest that the identified enhancers, which are mediated by FOXA1, can regulate *CYR61* expression to enhance the metastasis of colon cancer cells.

## Discussion

Enhancer regulation is an emerging mechanism of cancer progression [41, 42]. However, the functional mapping of cancer-specific enhancers is still in its early stages. Herein, we identify enhancers in the regulation of *CYR61* expression in colon cancer and the underlying mechanism. Our findings are as follows: (i) selectively active enhancers of *CYR61* are formed in colon cancer, but not in normal colon mucosa, that up-regulate *CYR61* expression to promote the migration of colon cancer cells; (ii) FOXA1 up-regulates the expression of *CYR61* by recruiting the lysine acetyltransferase CBP to the *CYR61* promoter and enhancer regions, increasing enhancer activity and forming a promoter-enhancer loop structure; and (iii) treatment with TPA and VP, compounds previously shown to up- and down-regulate *CYR61* expression, respectively, also respectively up- and down-regulate *CYR61* expression by affecting enhancer activity.

Active *CYR61* enhancers, which are enriched in H3K27Ac, are not present in normal colon tissue and cells, but are found in CRC tissue and colon cancer cell lines (Fig. 2a, b, Additional file 1: Figure S2A), indicating that *CYR61* enhancers are activated in cancer cells. A recent report of super-enhancers at the *c-MYC* oncogene locus, which are not active in normal colon cells but are active in HCT116 cells, represents another example of cancer-specific enhancers [43]. Moreover, different cancer cell lines show different enhancer regulation patterns, as reflected by one active enhancer (Enhancer3) in HCT116 cells, but two independent active enhancers (Enhancer1 and Enhancer3) in RKO cells. There was also a predicted Enhancer 2 (Fig. 2). However, cell experiments did not support Enhancer2 to be an active enhancer of *CYR61*. As Fig. 2c shows, Enhancer2 is decorated with low levels of H3K27ac and does not loop to the *CYR61* promoter (Fig. 2e). Moreover, its activity remains unchanged after TPA and VP stimulation (Fig. 5). Likewise, Enhancer1 in HCT116 cells is also decorated with low levels of H3K27ac (Additional file 1: Figure S2A) and does not form a loop with the *CYR61* promoter. Concomitantly, TF enrichment at Enhancer1 is also low (Additional file 1: Figure S3C, F).

Pioneer factor FOXA1 and acetyltransferase CBP play a strategic role in the regulation of *CYR61* expression. On one hand, both FOXA1 and CBP are needed to maintain H3K27ac enrichment at the *CYR61* promoter and enhancer regions. On the other hand, these two TFs are also required for the maintenance of the promoter-enhancer 3D structure. Decreasing FOXA1 binding reduced CBP enrichment at the enhancer regions. In contrast, decreasing CBP levels did not affect FOXA1 binding. These observations suggest that CBP is recruited after the binding of FOXA1, consistent with FOXA1 being a pioneer factor [44]. We, therefore, infer that FOXA1 functions as a pioneer factor to anchor to chromatin and recruit additional TFs, such as CBP, to establish the open state of chromatin and mediate promoter-enhancer looping.

Although H3K27ac [45], H3K4me1 [46] and H3K4me3 [47] have been reported to be associated with gene transcription and enhancer activity, in our case, H3K27ac is the critical epigenetic modification involved in *CYR61* regulation. In both the up-regulation of *CYR61* by TPA stimulation or down-regulation of *CYR61* by VP stimulation, only H3K27ac enrichment at *CYR61* promoter and enhancer regions shows statistical changes that parallel *CYR61* expression levels.

Our clinical data demonstrate that *CYR61* expression levels are up-regulated in primary colonic adenocarcinoma tissue and positively correlate with tumor grade. Migration experiments and mouse metastasis experiments show that CYR61 promotes colon cancer cells migration and metastasis. Similar observations have been documented in earlier studies [7]. By increasing enhancer activity, FOXA1 can positively regulate *CYR61* expression to promote colon cancer cell migration and metastasis.

Based on our current study, we present a schematic model to illustrate the role of enhancers in the regulation of *CYR61* expression (Fig. 7). In this model, abnormal enhancer activity (Enhancer3 in HCT116, and Enhancer1 and Enhancer3 in RKO cells) of *CYR61* occurs in CRC and up-regulates the expression of *CYR61*. Based on the crucial role of Mediator in the organization of genomic DNA topological structure [48] and a recent study showing that FOXA1 is essential for the recruitment of Mediator [49], we speculate that, under our conditions, the Mediator complex is likely to contribute to the role of FOXA1 and CBP in long-range looping. By mediating binding to the Mediator complex, FOXA1 and CBP bring the *CYR61* promoter and enhancers to the same transcriptional foci to enhance *CYR61* transcription in an active chromatin region. However, the molecular mechanisms by which FOXA1 is activated and induced to bind to DNA elements in specific colon cancer subtypes remain to be fully understood.

## Conclusions

In summary, our study reveals previously undocumented enhancers that are not active in normal colon mucosa, but become activated in CRC. This CRC-related selective enhancer regulation suggests that inhibition of recruitment of FOXA1 and/or CBP to enhancers and the promoter of *CYR61* may have therapeutic implications in CRC.

## Additional file

Additional file 1: **Figure S1.** Correlation of CYR61 expression of CRC tissues in IHC and clinicopathological parameters. **Figure S2.** Identification of *CYR61* enhancers. **Figure S3.** Enrichment of FOXA1 and CBP at target loci. **Figure S4.** TPA and VP regulate *CYR61* expression. **Table S1.** Primers sequences for real-time RT- qPCR. **Table S2.** Data for ChIP-seq and GRO-seq data downloaded from GEO. **Table S3.** Primers sequences for ChIP. **Table S4.** Sequences of siRNA oligonucleotide. **Table S5.** Plasmids and primers used in dual-luciferase reporter assays. **Table S6.** Primer sequences for 3C assay. **Table S7.** Histopathological features and clinical data of the patients. (DOCX 522 kb)

## **Abbreviations**

3C: chromosome conformation capture assay
CRC: colorectal cancer
CYR61: cysteine-rich 61
eRNAs: enhancer RNAs
FOXA1: forkhead box A1
GEO: Gene Expression Omnibus
H3K27ac: acetylated H3 lysine 27
H3K4me1: monomethylated H3 lysine 4
IHC: immunohistochemistry
qRT-PCR: quantitative real-time PCR
TFs: transcription factors
TPA: 12-O-tetradecanoyl phorbol-13-acetate
VP: verteporfin
WB: western blotting
