# Supplementary material for: Aberrant activation of CYR61 enhancers in colorectal cancer development
Source: J Exp Clin Cancer Res. 2019 May 22;38:213. doi: 10.1186/s13046-019-1217-9 (PMC6532222; doi:10.1186/s13046-019-1217-9)
Supplement: Supplementary file 1 — Figure S1. Correlation of CYR61 expression of CRC tissues in IHC and clinicopathological parameters. Figure S2. Identification of CYR61 enhancers. Figure S3. Enrichment of FOXA1 and CBP at target loci. Figure S4. TPA and VP regulate CYR61 expression. Table S1. Primers sequences for real-time RT- qPCR. Table S2. Data for ChIP-seq and GRO-seq data downloaded from GEO. Table S3. Primers sequences for ChIP. Table S4. Sequences of siRNA oligonucleotide. Table S5. Plasmids and primers used in dual-luciferase reporter assays. Table S6. Primer sequences for 3C assay. Table S7. Histopathological features and clinical data of the patients. (DOCX 522 kb) [file 13046_2019_1217_MOESM1_ESM.docx]

# Supplementary figures and tables

Aberrant activation of *CYR61* enhancers in colorectal cancer development

Lingzhu Xie^a^, Xuhong Song^a^, Hao Lin^b^, Zikai Chen^a^, Qidong Li^a^, Tangfei Guo^a^, Tian Xu^a^, Ting Su^a^, Man Xu^a^, Xiaolan Chang^a^, Long-kun Wang^c^, Bin Liang^a,1^, and Dongyang Huang^a,1^

^a^Department of Cell Biology and Genetics, Key Laboratory of Molecular Biology in High Cancer Incidence Coastal Chaoshan Area of Guangdong Higher Education Institutes, Shantou University Medical College, Shantou, 515041, China; ^b^Department of [Gastroenterology](D:\\AppData\\Roaming\\AppData\\Roaming\\Program Files (x86)\\Youdao\\Dict\\7.5.0.0\\resultui\\dict\\), Shantou Central Hospital, Shantou, 515041, China; ^c^Department of [Clinical](file:///D:\AppData\Roaming\AppData\Roaming\Program%20Files%20(x86)\Youdao\Dict\7.5.0.0\resultui\dict\) Laboratory, Jiujiang First People’s Hospital, Jiujiang, 332000, China

^1^ Author to whom correspondence should be addressed:

Dr. Dongyang Huang or Dr. Bin Liang;

Department of Cell Biology and Genetics, Shantou University Medical College, Complex Building, Room 602, No.22 Xinling Road, Shantou, Guangdong, China;

E-mail: [huangdy@stu.edu.cn](mailto:huangdy@stu.edu.cn) or [bliang@stu.edu.cn](mailto:bliang@stu.edu.cn)

# Supplementary figures


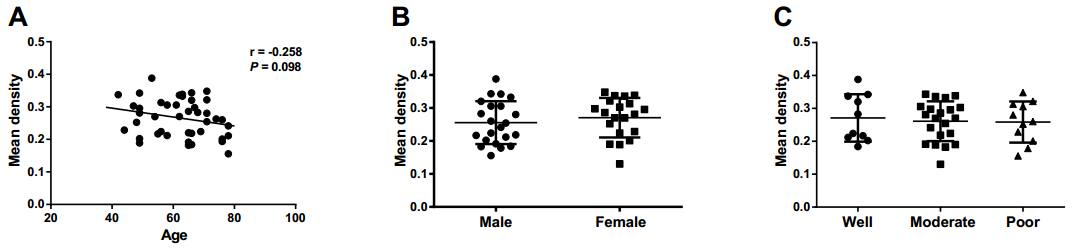


**Figure S1. Correlation of CYR61 expression of CRC tissues in IHC and clinicopathological parameters.** (A) The correlation between the mean density of CYR61 and patient ages. Significance was determined by Pearson correlation analysis. (B) Correlation between the mean density of CYR61 and patient sex, significance determined by the independent samples t-test. (C) Correlation between the mean density of CYR61 and tumor differentiation grades, significance determined by one-way ANOVA. Data are shown as mean ± S.D., n = 42.


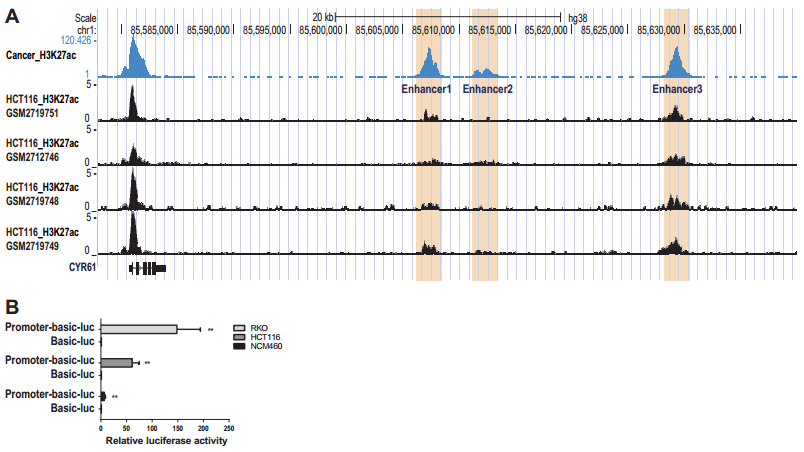


**Figure S2. Identification of *CYR61* enhancers.** (A) Enrichment of H3K27ac in HCT116 cells at the putative enhancer regions identified in different ChIP-seq data. H3K27ac showed higher enrichment encompassing Enhancer3. (B) Promoter relative luciferase activities in different cell lines, normalized to expression of Renilla luciferase from a co-transfected pRL-SV40 plasmid, tested by the independent samples t-test. Data are shown as mean ± S.D., n = 3. ***P* < 0.01.

#
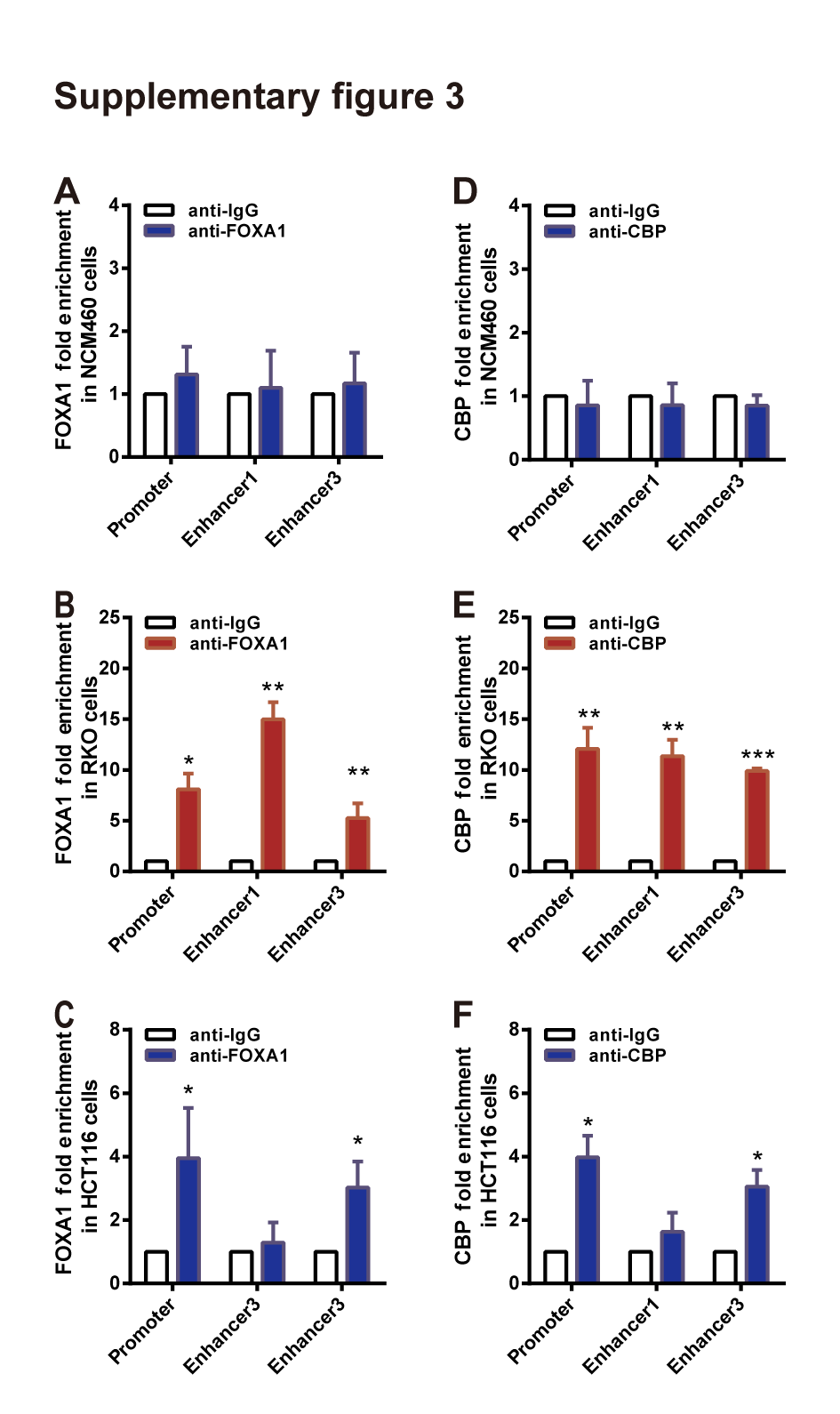


**Figure S3. Enrichment of FOXA1 and CBP at target loci.** (A) Enrichment of FOXA1 in NCM460. (B) Enrichment of FOXA1 in RKO cells. (C) Enrichment of FOXA1 in HCT116 cells. (D) Enrichment of CBP in NCM460 cells. (E) Enrichment of CBP in RKO cells. (F) Enrichment of CBP in HCT116 cells. Detected by ChIP-qPCR, tested by the independent samples t-test. Data are shown as mean ± S.D., n = 3. **P* < 0.05, ***P* < 0.01, ****P* < 0.001.


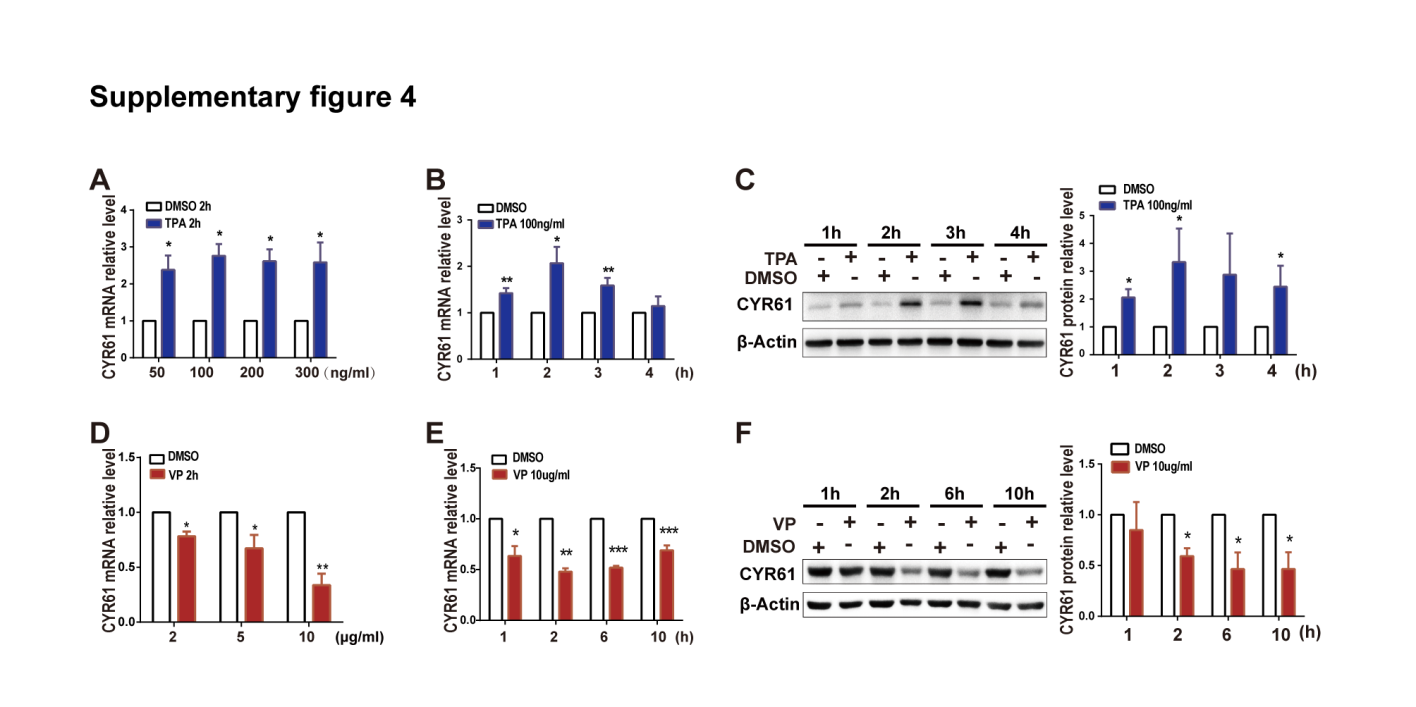


**Figure S4. TPA and VP regulate *CYR61* expression.** (A)~(C) TPA treatment of HCT116 cells. (A) *CYR61* mRNA levels were increased after treatment with various amounts of TPA for 2 h. (B) *CYR61* mRNA levels and (C) protein levels were increased after treatment with 100 ng/ml TPA for various times. (D)~(F) VP treatment in RKO cells. (D) *CYR61* mRNA levels showed dose-dependent decreases after treatment with various amounts of VP for 2 h. (E) *CYR61* mRNA levels and (F) protein levels were decreased after treatment with 10 μg/ml VP for various times. Detected by RT-qPCR and WB, tested by the independent samples t-test. Data are shown as mean ± S.D., n = 3. **P* < 0.05, ***P* < 0.01, ****P* < 0.001.

# Supplementary tables

**Table S1.** Primers sequences for real-time RT- qPCR

| Primer Names | Primer sequences |
| --- | --- |
| CYR61-F | 5’- ACCAATGACAACCCTGAGTG -3’ |
| CYR61-R | 5’- AAACATCCAGCGTAAGTAAACC -3’ |
| CYR61-eRNA-1-F | 5’- GCAGTCACCAATCTCAGTAGC -3’ |
| CYR61-eRNA-1-R | 5’- TCAAGCACATCCTCCAATAGC -3’ |
| CYR61-eRNA-2-F | 5’- ACTCAGCCCAGAACTCAAATC -3’ |
| CYR61-eRNA-2-R | 5’- GACGGAATACATTAGCCACATC -3’ |
| CYR61-eRNA-3-F | 5’- AGGCAGATGAAGTGACGAATGTG -3’ |
| CYR61-eRNA-3-R | 5’- CTGGCTCTTGTTGGATTTGTGTG -3’ |
| CBP-F | 5’- CACAGCCTCTCAGTCAACATC -3’ |
| CBP-R | 5’- CCTGGCGTAACTCCTCTGG -3’ |
| FOXA1-F | 5’- AGGAACTGTGAAGATGGAAGG -3’ |
| FOXA1-R | 5’- ATGTTGCCGCTCGTAGTC -3’ |
| β-actin-F | 5’- TTGGCAATGAGCGGTTCC -3’ |
| β-actin-R | 5’- AGACAGCACTGTGTTGGC -3’ |

**Table S2.** Data for ChIP-seq and GRO-seq data downloaded from GEO

| **No.** | **Category** | **Dataset** | **accession number** | **Run accession** | **Sample type** | **antibody** |
| --- | --- | --- | --- | --- | --- | --- |
| 1 | ChIP-seq | GSM915331 | SRX142118 | SRR486244 | Normal Colon Tissue | H3K27ac |
| 2 | ChIP-seq | GSM883681 | SRX124705 | SRR424634 | Colon Cancer Tissue | H3K27ac |
| 3 | ChIP-seq | GSM956020 | SRX157638 | SRR518297 | Normal Colon Tissue | H3K4me1 |
| 4 | ChIP-seq | GSM883660 | SRX124684 | SRR424613 | Colon Cancer Tissue | H3K4me1 |
| 5 | ChIP-seq | GSM956024 | SRX157642 | SRR518490 | Normal Colon Tissue | H3K4me3 |
| 6 | ChIP-seq | GSM883688 | SRX124712 | SRR424641 | Colon Cancer Tissue | H3K4me3 |
| 7 | ChIP-seq | GSM2719751 | SRX3043701 | SRR5876185 | HCT116 | H3K27ac |
| 8 | ChIP-seq | GSM2712746 | SRX3024371 | SRR5855055 | HCT116 | H3K27ac |
| 9 | ChIP-seq | GSM2719748 | SRX3043698 | SRR5876182 | HCT116 | H3K27ac |
| 10 | ChIP-seq | GSM2719749 | SRX3043699 | SRR5876183 | HCT116 | H3K27ac |
| 11 | GRO-seq | GSM1124062 | SRX266757 | SRR828695 | HCT116 | —— |

**Table S3.** Primers sequences for ChIP

| **Primer Names** | **Primer sequences** |
| --- | --- |
| CYR61-promoter-F | 5’- TCCGAACACGCCTCTTTGAAGTC -3’ |
| CYR61-promoter-R | 5’- AGCGAAGGTGAGAGGCAAGTTATC -3’ |
| Enhancer-1-F | 5’- AGAAGGCAGCACAGCAGAG -3’ |
| Enhancer-1-R | 5’- CAGTGACCTCAGACAATAAGAACC -3’ |
| Enhancer-2-F | 5’- CCTGGCTTTCACCTTTCTTG -3’ |
| Enhancer-2-R | 5’- TGTCTTATTCAGCATTCTACTACTC -3’ |
| Enhancer-3-F | 5’- CCGCTGCTGCCCGTAAAG -3’ |
| Enhancer-3-R | 5’- AAGTCACCAGCCAAGGAAGTC -3’ |
| NC-F | 5’- GATGCTCTCCTTGCCACTTAG -3’ |
| NC-R | 5’- GATGGACTCTGTAAGAAGGAAGC -3’ |

**Table S4.** Sequences of siRNA oligonucleotide

| **Names** | **Sequences** |
| --- | --- |
| Si control sense | 5’- UUCUCCGAACGUGUCACGUTT -3’ |
| Si control antisense | 5’- ACGUGACACGUUCGGAGAATT -3’ |
| Si CYR61 sense | 5’- GCAUCCUAUACAACCCUUUTT -3’ |
| Si CYR61 antisense | 5’- AAAGGGUUGUAUAGGAUGCTT -3’ |
| Si CBP sense | 5’- CCAUUUCUCCUUCCCGAAUTT -3’ |
| Si CBP antisense | 5’-AUUCGGGAAGGAGAAAUGGTT -3’ |
| Si FOXA1 sense | 5’- GCCAUGAACAGCAUGACUGTT -3’ |
| Si FOXA1 antisense | 5’- CAGUCAUGCUGUUCAUGGCTT -3’ |

**Table S5.** Plasmids and primers used in dual-luciferase reporter assays

| **Names** | **Inserted Sequences** | **Inserted site** | **Primer-F** | **Primer-R** |
| --- | --- | --- | --- | --- |
| pGL3-basic |  |  |  |  |
| pGL3-basic-CYR61promoter | chr1: 85579867-85581178 | KpnI-MluI | 5’-CGGGGTACCGGCTGGAACTAAAGTGGGAAC -3’ | 5’- CGACGCGTAGTGCTTAAAGAAACGGCTACC -3’ |
| pGL3-basic-CYR61promoter-enh1+ | chr1: 85605857-85608698 | SalI | 5’- ACGCGTCGACGCAAGAATAGAGGTAAAGAC -3’ | 5’- ACGCGTCGACGTCAAAGGAAGAACTAAAGC -3’ |
| pGL3-basic-CYR61promoter-enh1- |  |  |  |  |
| pGL3-basic-CYR61promoter-enh2+ | chr1: 85610909-85613823 | SalI | 5’- ACGCGTCGACAATGGTAACTACTCTCCTG -3’ | 5’- ACGCGTCGACGTAACTTTGTCATCATTCTTTG -3’ |
| pGL3-basic-CYR61promoter-enh2- |  |  |  |  |
| pGL3-basic-CYR61promoter-enh3+ | chr1: 85628027-85630656 | SalI | 5’- ACGCGTCGACTCCTGGCTGTCTTCCTTACC -3’ | 5’- ACGCGTCGACTAACTGGCTGATTACTGAACCTC -3’ |
| pGL3-basic-CYR61promoter-enh3- |  |  |  |  |
| pGL3-basic-CYR61promoter-enhNC | chr1: 85635005-85637877 | SalI | 5’- ACGCGTCGACCACAGAACGGCACAGTCAAG -3’ | 5’- ACGCGTCGACTCACCTCATCCAGCCTTCATAG -3’ |

**Table S6.** Primer sequences for 3C assay

| **Primer Names** | **Paired primer** | **Forward Primer sequences** |
| --- | --- | --- |
| Promoter1 |  | 5’- AAGAGATAATAAGATTAGTTGGACAG -3’ |
| Promoter2 |  | 5’- TGGGGTTCTACAGTCGTAAAAG -3’ |
| HindⅢ-1F | Promoter1 | 5’- TATTACAAGAAATACAGAATGTCAAAC -3’ |
| HindⅢ-2F | Promoter1 | 5’- CTCCCAGCACAAAACCTAG -3’ |
| HindⅢ-3F | Promoter2 | 5’- CGGATGATACTTTCCCTTTGTG -3’ |
| HindⅢ-4F | Promoter1 | 5’- ATGGGTATGAGTCTAAGCAATC -3’ |
| HindⅢ-5F | Promoter2 | 5’- AAAGAGAGCAGAGATGAGAAACAC -3’ |
| HindⅢ-6F | Promoter1 | 5’- CCCAAAGGCAGGATTATTATAC -3’ |
| HindⅢ-7F | Promoter1 | 5’- AGCAGTAATATAAAGGCAATAACAATTC -3’ |
| HindⅢ-8F | Promoter1 | 5’- GCAAGGCTGGAGTTGGAC -3’ |
| HindⅢ-9F | Promoter2 | 5’- GGGAGATGCCTTTGCTTTG -3’ |
| HindⅢ-10F | Promoter1 | 5’- AGGACAAATGGTGGATAGCG -3’ |
| HindⅢ-11F | Promoter1 | 5’- GATGCTCTCCTTGCCACTTAG -3’ |
| ERCC3-1F^†^ |  | 5’- GTGTAACAGGAAAGAAAGCAAATG -3’ |
| ERCC3-1R^†^ | ERCC3-1F | 5’- GTTTGCCTCCCAGACATCAG -3’ |
| ERCC3-2F | ERCC3-1F | 5’- TACTGGCTATCCTCAAGTTACC -3’ |
| β-Actin-3C-F^*†^ |  | 5’- AGGAAGGAAGGCTGGAAGAG -3’ |
| β-Actin-3C-R^*†^ | β-actin-3C-F | 5’- TGCGTGACATTAAGGAGAAGC -3’ |
| ^*^ used for loading quantity determination;  ^†^ used for digestion efficiency determination. The others were for 3C-qPCR of enhancers and promoter interaction. | | |

**Table S7.** Histopathological features and clinical data of the patients

| **Case No.** | **Histologic type** | **Tumor stage** | **Gender**^*^ | **Age  (year)** | **Tumor stage** ^†^ | **Differentiation grade** ^‡^ | **Mean density** ^§^ | |
| --- | --- | --- | --- | --- | --- | --- | --- | --- |
|  |  |  |  |  |  |  | **normal** | **cancer** |
| 1 | Adenocarcinoma | T_4_N_2_M_0_ | 2 | 76 | 3 | 3 | 0.1754 | 0.2012 |
| 2 | Adenocarcinoma | T_3_N_0_M_0_ | 1 | 71 | 2 | 3 | 0.1581 | 0.2803 |
| 3 | Adenocarcinoma | T_4_N_1_M_1_ | 2 | 49 | 4 | 2 | 0.1468 | 0.2957 |
| 4 | Adenocarcinoma | T_4_N_1_M_1_ | 2 | 48 | 4 | 3 | 0.1918 | 0.2522 |
| 5 | Adenocarcinoma | T_4_N_2_M_1_ | 2 | 44 | 4 | 3 | 0.2849 | 0.2285 |
| 6 | Adenocarcinoma | T_4_N_2_M_1_ | 1 | 66 | 4 | 2 | 0.1980 | 0.3432 |
| 7 | Adenocarcinoma | T_4_N_2_M_0_ | 2 | 56 | 3 | 3 | 0.0865 | 0.3130 |
| 8 | Adenocarcinoma | T_3_N_2_M_0_ | 1 | 71 | 3 | 2 | 0.1368 | 0.2546 |
| 9 | Adenocarcinoma | T_3_N_1_M_0_ | 2 | 67 | 3 | 2 | 0.1870 | 0.2977 |
| 10 | Adenocarcinoma | T_2_N_0_M_0_ | 2 | 76 | 1 | 2 | 0.1937 | 0.1901 |
| 11 | Adenocarcinoma | T_3_N_1_M_1_ | 1 | 63 | 4 | 2 | 0.1197 | 0.3328 |
| 12 | Adenocarcinoma | T_4_N_2_M_1_ | 1 | 53 | 4 | 1 | 0.1059 | 0.3881 |
| 13 | Adenocarcinoma | T_4_N_1_M_0_ | 2 | 42 | 3 | 1 | 0.1843 | 0.3375 |
| 14 | Adenocarcinoma | T_4_N_0_M_1_ | 1 | 66 | 4 | 1 | 0.1335 | 0.3202 |
| 15 | Adenocarcinoma | T_3_N_1_M_0_ | 1 | 49 | 3 | 1 | 0.1330 | 0.2022 |
| 16 | Adenocarcinoma | T_4_N_1_M_0_ | 2 | 71 | 3 | 3 | 0.1242 | 0.3213 |
| 17 | Adenocarcinoma | T_2_N_0_M_0_ | 2 | 54 | 1 | 2 | 0.1472 | 0.2700 |
| 18 | Adenocarcinoma | T_3_N_1_M_0_ | 2 | 62 | 3 | 2 | 0.1167 | 0.2702 |
| 19 | Adenocarcinoma | T_3_N_0_M_0_ | 2 | 65 | 2 | 2 | 0.1818 | 0.1301 |
| 20 | Adenocarcinoma | T_4_N_0_M_1_ | 2 | 65 | 4 | 2 | 0.1140 | 0.2860 |
| 21 | Adenocarcinoma | T_4_N_1_M_1_ | 1 | 61 | 4 | 2 | 0.1528 | 0.3057 |
| 22 | Adenocarcinoma | T_2_N_0_M_0_ | 1 | 58 | 1 | 1 | 0.1826 | 0.2119 |
| 23 | Adenocarcinoma | T_4_N_0_M_0_ | 2 | 71 | 2 | 3 | 0.1797 | 0.3483 |
| 24 | Adenocarcinoma | T_3_N_1_M_0_ | 2 | 49 | 3 | 2 | 0.1652 | 0.1889 |
| 25 | Adenocarcinoma | T_4_N_1_M_0_ | 1 | 78 | 3 | 3 | 0.1106 | 0.1554 |
| 26 | Adenocarcinoma | T_4_N_0_M_0_ | 1 | 58 | 2 | 3 | 0.1502 | 0.3057 |
| 27 | Adenocarcinoma | T_4_N_0_M_0_ | 2 | 47 | 2 | 2 | 0.1608 | 0.3028 |
| 28 | Adenocarcinoma | T_2_N_0_M_0_ | 2 | 56 | 1 | 2 | 0.1210 | 0.2241 |
| 29 | Adenocarcinoma | T_3_N_2_M_0_ | 1 | 78 | 3 | 2 | 0.1335 | 0.2416 |
| 30 | Adenocarcinoma | T_3_N_0_M_0_ | 1 | 49 | 2 | 1 | 0.2148 | 0.3424 |
| 31 | Adenocarcinoma | T_4_N_0_M_0_ | 1 | 55 | 2 | 1 | 0.1276 | 0.2171 |
| 32 | Adenocarcinoma | T_3_N_0_M_0_ | 1 | 78 | 2 | 2 | 0.2110 | 0.1825 |
| 33 | Adenocarcinoma | T_4_N_0_M_0_ | 1 | 66 | 2 | 2 | 0.1557 | 0.2185 |
| 34 | Adenocarcinoma | T_4_N_0_M_0_ | 1 | 76 | 2 | 3 | 0.2109 | 0.2601 |
| 35 | Adenocarcinoma | T_4_N_0_M_0_ | 1 | 69 | 2 | 1 | 0.1252 | 0.2241 |
| 36 | Adenocarcinoma | T_4_N_0_M_0_ | 1 | 66 | 2 | 1 | 0.1442 | 0.1841 |
| 37 | Adenocarcinoma | T_4_N_0_M_0_ | 1 | 68 | 2 | 1 | 0.1177 | 0.2826 |
| 38 | Adenocarcinoma | T_4_N_0_M_0_ | 2 | 63 | 4 | 2 | 0.1630 | 0.3386 |
| 39 | Adenocarcinoma | T_4_N_0_M_0_ | 1 | 65 | 2 | 2 | 0.1144 | 0.1913 |
| 40 | Adenocarcinoma | T_4_N_0_M_0_ | 2 | 62 | 2 | 2 | 0.1766 | 0.3360 |
| 41 | Adenocarcinoma | T_4_N_1_M_1_ | 2 | 49 | 4 | 2 | 0.1468 | 0.2811 |
| 42 | Adenocarcinoma | T_3_N_0_M_0_ | 1 | 65 | 2 | 3 | 0.2203 | 0.1786 |

^*^Gender was assigned as follows: 1, male; 2, female;

^†^Tumor stage was assigned as follows: I,1; IIA-C,2; IIIA-C,3; IVA-B, 4;

^‡^Differentiation grade was scored as follows: 1, well; 2, moderately; and 3, poorly differentiated;

^§^Mean density = IOD / Area (measured by IPP 6.0). The average mean density for all of five random fields at 100× magnification was used.
